# High amplification of FGFR1 gene is a delayed poor prognostic factor in early stage ESCC patients

**DOI:** 10.18632/oncotarget.20215

**Published:** 2017-08-12

**Authors:** Qi Song, Yalan Liu, Dongxian Jiang, Haixing Wang, Jie Huang, Yifan Xu, Akesu Sujie, Haiying Zeng, Chen Xu, Yingyong Hou

**Affiliations:** ^1^ Department of Pathology, Zhongshan Hospital, Fudan University, Shanghai 200032, P. R. China; ^2^ Department of Pathology, School of Basic Medical Sciences & Zhongshan Hospital, Fudan University, Shanghai 200032, P. R. China

**Keywords:** *FGFR1* high amplification, clinical stage, disease free survival time, prognostic marker, ESCC

## Abstract

Amplification of the fibroblast growth factor receptor 1 (*FGFR1*) is believed to predict response to FGFR inhibitors. The aim of this study was to investigate the frequency and the prognostic impact of *FGFR1* amplification in patients with resected esophageal squamous cell carcinoma (ESCC) by using fluorescent in situ hybridization. Microarrayed paraffin embedded blocks were constructed, and the cohort of tissues came from 506 patients with ESCC. *FGFR1* high amplification (*FGFR1*^*high*^) was defined by an *FGFR1*/centromere 8 ratio of ≥ 2.0, or average number of *FGFR1* signals/tumor cell nucleus ≥ 6.0, or percentage of tumor cells containing ≥ 15 *FGFR1* signals, or large cluster in ≥ 10% of cancer cells. *FGFR1* low amplification was defined by ≥ 5 *FGFR1* signals in ≥ 50% of cancer cells. Kaplan-Meier curves with log-rank tests and Cox proportional hazards model were used to analyze patients’ survival. Among 506 patients, high amplification, low amplification, and disomy were detected in 8.7%, 3.6% and 87.7%, respectively. In general, the *FGFR1*^*high*^ group trended towards worse disease-free survival (DFS) and overall survival (OS) compared to the *FGFR1* low amplification/disomy (*FGFR1*^*low/disomy*^) group (DFS, *P*=0.108; OS, *P*=0.112), but this trend was amplified for patients with DFS ≥ 30 months (DFS, *P*=0.009; OS, *P*=0.007). Furthermore, when patients were stratified into stage I-II and stage III-IV, the *FGFR1*^*high*^ group directly presented with adverse DFS and OS than the *FGFR1*^*low/disomy*^ group in stage I-II patients (DFS, *P*=0.019; OS, *P*=0.034), especially with DFS ≥ 30 months (DFS, *P*=0.002; OS, *P*=0.001). However, for patients in stage III-IV, *FGFR1*^*high*^ had no effect on prognosis regardless of DFS time. *FGFR1*^*high*^ occurs in a minority of ESCC, and it predicts delayed poor prognosis in stage I and II ESCC patients.

## INTRODUCTION

Esophageal cancer (EC) is the sixth leading cause of cancer-related mortality worldwide resulting in more than 400,000 deaths annually [1]. A lack of effective chemotherapeutic approaches available to treat patients with EC combined with the fact that many EC patients are diagnosed at advanced stages both contribute to the poor prognosis of this disease [2]. Based on histologic criteria, EC is separated into two major types: esophageal squamous cell cancer (ESCC) and esophageal adenocarcinoma (EAC). ESCC accounts for approximately 90% of EC worldwide [3], which is the main subtype in China and ESCC is the third most commonly diagnosed cancer among men, while the fifth among women [4].

In recent years, studies that comprehensively characterized the genomic landscape of ESCC and EAC have led to an important understanding of the genetic basis of EC and identified genes associated with the pathogenesis of the specific EC subtypes [5-9]. EAC and ESCC represent distinct disease entities, which may benefit from different therapeutic strategies. Despite advances in personalized treatment of EAC [10, 11], effective targeted therapies for ESCC have remained elusive. Fibroblast growth factor receptor 1 (*FGFR1*) amplification is one of the most promising findings in ESCC genomic studies due to the availability of FGFR inhibitors and its association with response to FGFR inhibitor treatment [12, 13].

The FGFR tyrosine kinase family consists four kinases: *FGFR1*, *FGFR2*, *FGFR3*, and *FGFR4* and the ligands comprise 22 family members (fibroblast growth factors, FGFs). FGFRs share structural homology with many pharmacologic therapeutic targets, such as vascular endothelial growth factor receptors (VEGFRs) and platelet-derived growth factor receptors (PDGFRs) [14]. Receptor activation by FGFs initiates a series of intracellular events that activates major survival and proliferative signal pathways, and then regulate many biologic processes including the wound repair, formation of new blood vessels, and embryonic development [15]. More recently, increasing evidence demonstrated that FGFRs play crucial roles in cancer development. FGFRs are deregulated by amplification, point mutation, or translocation and amplification is the most common deregulation form in multiple cancer types [16-18]. Amplification of *FGFR1* has been reported in 13%-22% squamous cell lung cancer [13, 19, 20], 20% breast cancer [12, 21], 10%-17% head and neck squamous cell carcinoma [22, 23], and 26.9% malignant peripheral nerve sheath tumor [24]. *FGFR1* amplification induced a strong *FGFR1* dependency that could be exploited therapeutically, and *in vivo* studies have demonstrated inhibition of the *FGFR1* pathway with FGFR inhibitors that led to significant tumor shrinkage [13, 25], and translational clinical trials are undertaken [26].

As the significant clinical value of *FGFR1*, the prevalence and the prognostic value of *FGFR1* amplification in ESCC is urgently needed to explore. Some researchers have reported that *FGFR1* amplification rate, ranging from 6% to 9.7%[27-29]. However, the prognostic value of *FGFR1* amplification is not consistent in different studies. Kim et al [27] recently reported high *FGFR1* amplification is an independent poor prognostic factor and a potential therapeutic target in ESCC. In another study [28] on Caucasian patients, there was no association between *FGFR1* amplification status and clinical outcome. Therefore, further detailed analysis is needed to investigate the prognostic significance of *FGFR1* amplification in ESCC.

In present study, we analyzed *FGFR1* amplification status in 506 ESCC patients with surgically resected and searched for correlations between *FGFR1* amplification and clinicopathological parameters. We meticulously explored the prognostic value of *FGFR1* amplification in these patients with the purpose of precisely predicting patients’ outcome.

## RESULTS

### Patient characteristics

**Figure 1:**
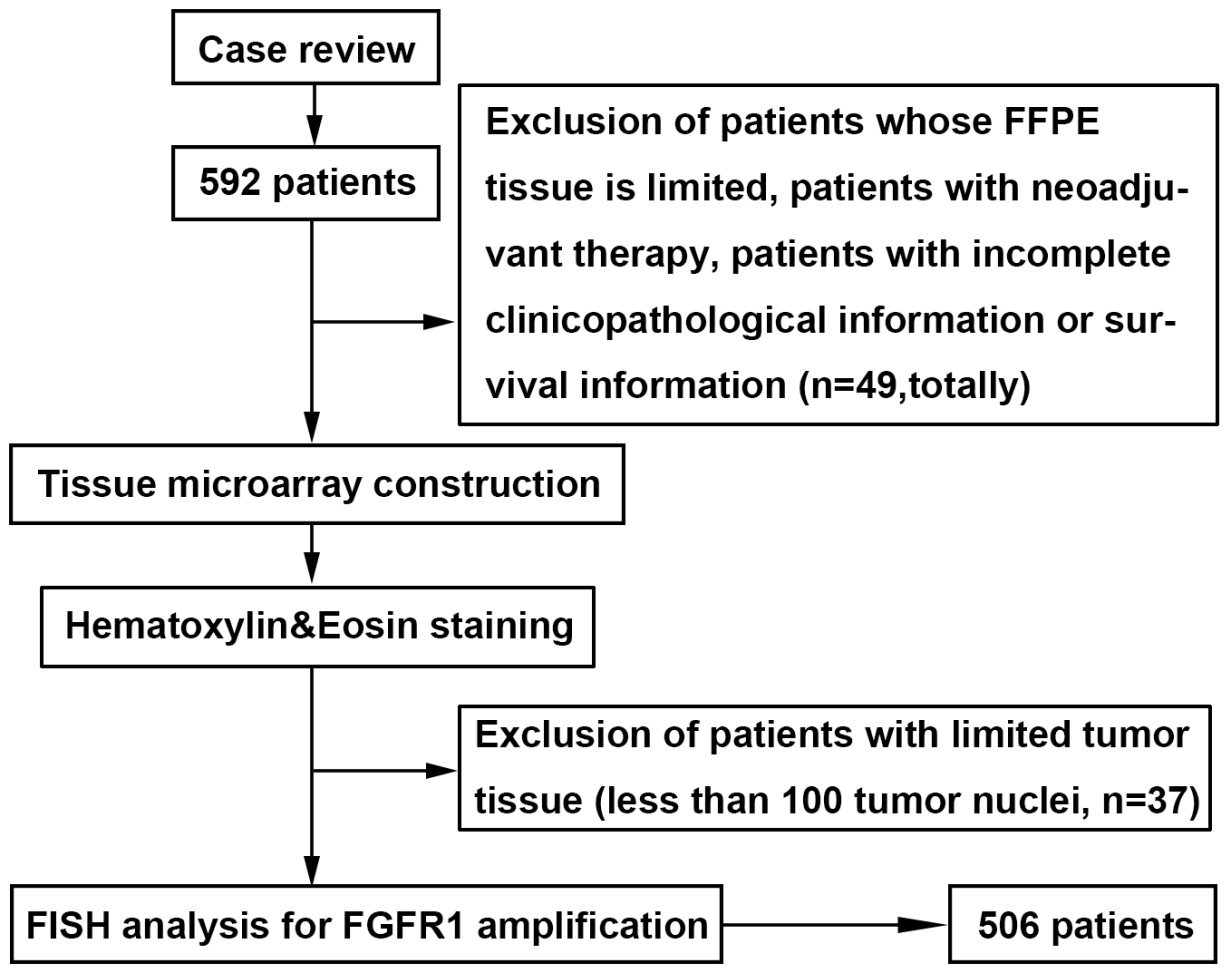
Patients and sample selection flow chart Cases with ESCC were identified retrospectively and re-reviewed. Patients whose paraffin-embedded tissue is limited, patients with neoadjuvant therapy, and those with incomplete clinicopathological information or survival information were excluded. Representative tissue blocks were selected, Tissue microarray (TMA) construction was undertaken, and hematoxylin-eosin staining was performed. Patients with limited tumor tissue (less than 100 tumor nuclei) in TMA were excluded. Fluorescent in situ hybridization for *FGFR1* was undertaken. The final cohort of ESCC patients consisted of 506 cases.

A total of 506 ESCC patients who underwent curative esophagectomy were enrolled (Figure 1) in our analysis and the clinical characteristics were listed in Table 1. There were 415 males and 91 females with a median age of 61.2 years (range 34-83). By anatomic site, 29 were in the upper esophagus, 238 in the middle and 239 in the lower area. A total of 111 tumors had invaded to the mucous layer or submucosa, 224 to the muscularis propria and 171 to the adventitia. Most of the tumor differentiation was grade II (55.7%), 40.1% was grade III, and only 4.2% was grade I. A total of 183 tumors were examined with nerve infiltration, 110 with vessel involvement, 244 with lymph node metastases, and 59 with distant metastases.

**Table 1:** Clinicopathological characteristics of the 506 patients with ESCC

Characteristics		All patients			Patients with Stage I-II disease				Patients with DFS time≥30months				Patients with DFS time≥30months in stage I-II			
		No.	%		No.		%		No.		%		No.		%	
Total		506	100.0		301		100.0		259		100.0		201		100.0	
Gender																
Male		415	82.0		236		78.4		205		79.2		154		76.6	
Female		91	18.0		65		21.6		54		20.8		47		23.4	
Age																
<60		215	42.5		121		40.2		117		45.2		89		44.3	
≥60		291	57.5		180		59.8		142		54.8		112		55.7	
Tumor site																
Upper		29	5.7		20		6.6		15		5.8		14		7.0	
Middle		238	47.0		158		52.5		124		47.9		106		52.7	
Low		239	47.2		123		40.9		120		46.3		81		40.3	
Differentiation																
Well		21	4.2		15		5.0		13		5.0		12		6.0	
Moderate		282	55.7		182		60.5		149		57.5		123		61.2	
Poor		203	40.1		104		34.6		97		37.5		66		32.8	
Clinical stage																
I+II		301	59.5						201		77.6					
III+IV		205	40.5						58		22.4					
Invasive depth																
I		111	21.9		106		35.2		76		29.3		73		36.3	
II		224	44.3		126		41.9		104		40.2		84		41.8	
III		171	33.8		69		22.9		79		30.5		44		21.9	
Lymph node metastasis																
No		262	51.8		250		83.1		178		68.7		177		88.1	
Yes		244	48.2		51		16.9		81		31.3		24		11.9	
Distant metastasis																
No		447	88.3		275		91.4		249		96.1		196		97.5	
Yes		59	11.7		26		8.6		10		3.9		5		2.5	
Vessel involvement																
No		396	78.3		268		89.0		209		80.7		177		88.1	
Yes		110	21.7		33		11.0		50		19.3		24		11.9	
Nerve involvement																
No		323	63.8		226		75.1		183		70.7		152		75.6	
Yes		183	36.2		75		24.9		76		29.3		49		24.4	
Necrosis																
No		353	69.8		225		74.8		185		71.4		152		75.6
Yes		153	30.2		76		25.2		74		28.6		49		24.4
Smoking																
No		309	61.1		202		67.1		165		63.7		136		67.7
Yes		197	38.9		99		32.9		94		36.3		65		32.3
FGFR1 amplification																
High		44	8.7		20		6.6		20		7.7		10		5.0
Low		18	3.6		8		2.7		9		3.5		7		3.5
Disomy		444	87.7		273		90.7		230		88.8		184		91.5

Invasive depth I, tumors had invaded to the mucous layer or submucosa; II, to the muscularis propria; III, to or beyond the adventitia.

### *FGFR1* amplification status and clinicopathological features

**Figure 2:**
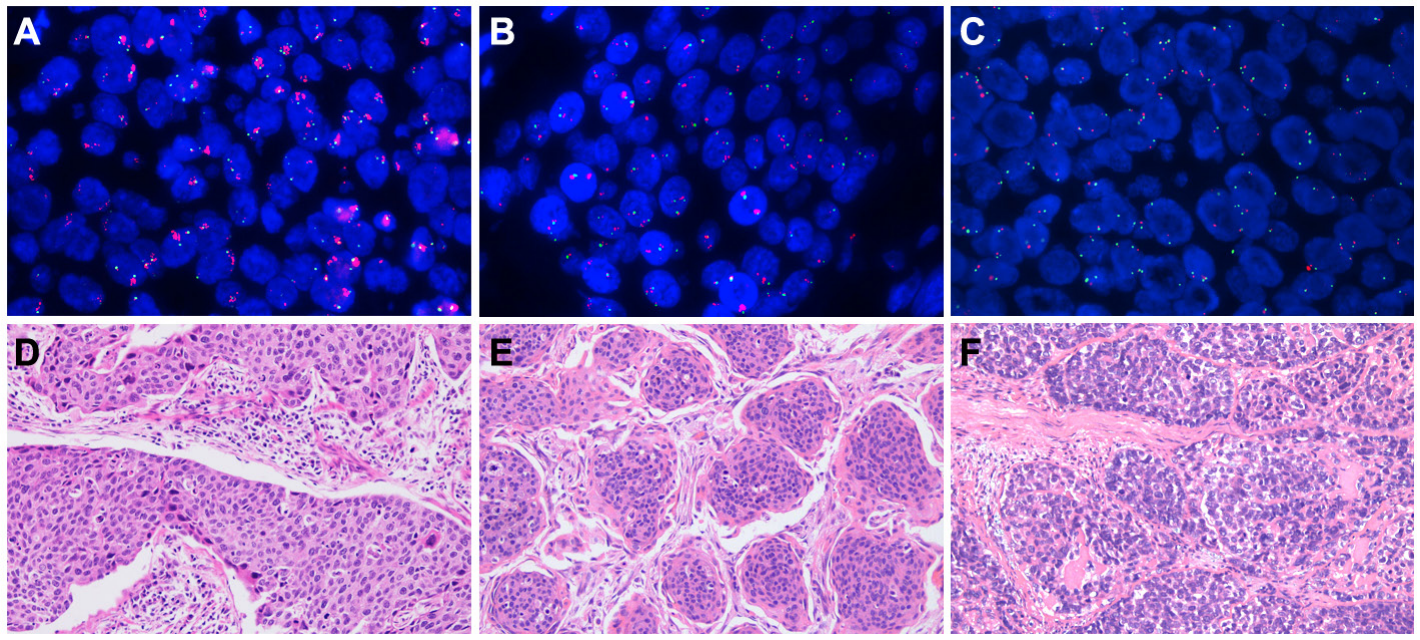
Fibroblast growth factor receptor 1 (*FGFR1*) amplification assessed by fluorescent in situ hybridization and the corresponding hematoxylin-eosin (HE) staining The *FGFR1* gene was labeled in red and the CEN8 reference probe in green. **(A)** high *FGFR1* amplification; **(B)** low *FGFR1* amplification; **(C)** disomy; **(D)** the corresponding HE staining of high *FGFR1* amplification; **(E)** the corresponding HE staining of low *FGFR1* amplification; **(F)** the corresponding HE staining of disomy.

Based on a previous study [30], patients were classified into three groups by using prespecified criteria for the *FGFR1* gene copy number and *FGFR1*/ centromere 8 (CEN8) ratio. Among a total of 506 patients, 44 (8.7%) were high *FGFR1* amplification, 18 (3.6%) were low *FGFR1* amplification, and 444 (87.7%) were disomy (Figure 2; Table 1). The median *FGFR1* gene copy number per nucleus and the mean *FGFR1*/CEN8 ratio in all patients were 2.45 (range, 0 to 12.25 copies per nucleus) and 1.21 (range, 0 to 4.64). The median *FGFR1* gene copy number was 6.57 (range, 3.19 to 12.25) in high amplification, 3.91 (range, 3.33 to 5.92) in low amplification, and 1.98 (range 0 to 4.20) in disomy group. The mean *FGFR1*/ CEN8 ratio was 3.75 (range 0.89 to 4.64), 1.49 (range, 1.12 to 1.90), and 0.95 (range, 0 to 1.75) in high, low and disomy group, respectively. Of 44 high *FGFR1* amplified tumors, 5 cases (11.4%) only satisfied the criterion of *FGFR1*/ CEN8 ratio is ≥ 2.0, 7 cases (15.9%) only satisfied the criterion of an average number of *FGFR1* signal per nucleus ≥6.0, and 26 cases (59.1%) only satisfied the criterion of percentage of tumor cells containing ≥ 15 *FGFR1* signals or large clusters in ≥ 10% cells. And only one case (2.3%) satisfied the criteria of *FGFR1*/CEN8 ratio is ≥ 2.0 and an average number of *FGFR1* signal per nucleus ≥6.0. Five cases (11.4%) satisfied all three criteria for *FGFR1* amplification.

Associations between *FGFR1* amplification and clinicopathological characteristics for the 506 patients were shown in Table 2. *FGFR1* amplification status correlated with clinical stage (*P*=0.047), lymph node metastasis (*P*=0.032), and necrosis (*P*=0.008). And high FGFR1 amplification is positively correlated with advanced stage, positive lymph node metastasis, and necrosis. There was no significant difference between the *FGFR1*^*high*^ and *FGFR1*^*low/disomy*^ group regarding gender, age, tumor site, invasive depth, distant metastasis, differentiation, vessel involvement, nerve involvement, and smoking.

**Table 2:** Association between FGFR1-FISH results and the clinicopathlolgical characteristics in the cohort of 506 ESCC patients.

	FGFR1-FISH results				
Characteristics	High amplification		Low amplification/Disomy		P
	No.	%	No.	%	
Total	44	8.7	462	91.3	
Gender					0.432
Male	38	9.2	377	90.8	
Female	6	6.6	85	93.4	
Age					0.677
<60	20	9.3	195	90.7	
≥60	24	8.2	267	91.8	
Tumor site					0.939
Upper	2	6.9	27	93.1	
Middle	21	8.8	217	91.2	
Low	21	8.8	218	91.2	
Differentiation					0.178
Well	0	0	21	100.0	
Moderate	22	7.8	260	92.2	
Poor	22	10.8	181	89.2	
Clinical stage					0.047*
I+II	20	6.6	281	93.4	
III+IV	24	11.7	181	88.3	
Invasive depth					0.167
I	6	5.4	105	94.6	
II	18	8.0	206	92.0	
III	20	11.7	151	88.3	
Lymph node metastasis					0.032*
No	16	6.1	246	93.9	
Yes	28	11.5	216	88.5	
Distant metastasis					0.358
No	37	8.3	410	91.7	
Yes	7	11.9	52	88.1	
Vessel involvement					0.352
No	32	8.1	364	91.9	
Yes	12	10.9	98	89.1	
Nerve involvement					0.180
No	24	7.4	299	92.6	
Yes	20	10.9	163	89.1	
Necrosis					0.008*
No	23	6.5	330	93.5	
Yes	21	13.7	132	86.3	
Smoking					0.491
No	29	9.4	280	90.6	
Yes	15	7.6	182	92.4	

Invasive depth I, tumors had invaded to the mucous layer or submucosa; II, to the muscularis propria; III, to or beyond the adventitia.

*P-value derived from χ^2^test.

### Survival outcomes in the cohort of ESCC patients

With a median follow-up time of 35 months (range 2-102 months), the 5-year DFS and OS rate for all patients were 44.6 % and 45%. Mean and median times to DFS were 55.5 and 36.0months. Mean and median times to OS were 59.2 and 42.0 months. The 5-year DFS rate according to clinical stages were 60.9% in stage I-II, and 20.3% in stage III-IV patients. The 5-year OS rate was 61.1% for stage I-II, and 21.0% for stage III-IV patients.

**Figure 3:**
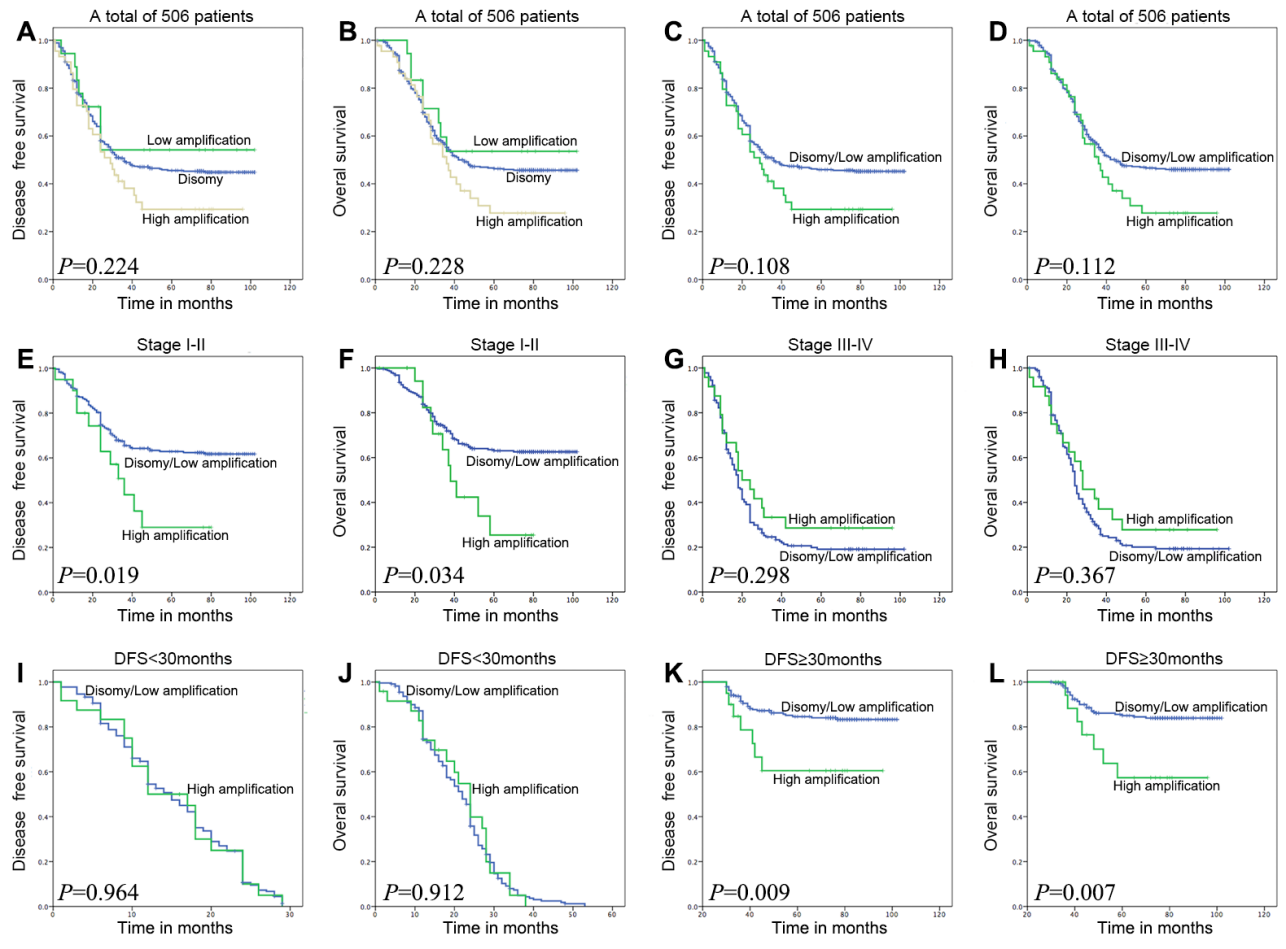
Survival analysis on the cohort of ESCC patients, on the basis of clinical stage, and on the basis of DFS time 30 months as a group-dividing value To the whole cohort of patients, the *FGFR1* amplification status has no correlation with DFS and OS time no matter in three groups or in two groups **(A, B, C, and D)**. In patients with stage I-II, *FGFR1*^*high*^ was associated with adverse DFS and OS (**E and F**; *P*=0.019 or 0.034). In DFS≥30 months group, *FGFR1*^high^ did represent poor DFS and OS (**K and L**; *P*=0.009 or 0.007). However, in patients with stage III-IV disease or DFS<30 months group, high *FGFR1* amplification was not associated with DFS and OS (**G, H, I and J**; *P*=0.298, 0.367, 0.964 or 0.912).

Kaplan-Meier curves with a log rank test for DFS and OS were undertaken to assess the possible association between ESCC *FGFR1* amplification and patient survival. As shown in Figure 3A and 3B, the estimated mean DFS times and OS times were not significantly different in three *FGFR1* groups, which was consistent with a previous report [27]. As the low amplification group has a better DFS and OS trend (DFS, *P*=0.118; OS, *P*=0.122) than *FGFR1*^*high*^ group and has no statistical difference (DFS, *P*=0.521; OS, *P*=0.504) with the disomy group, we categorized total patients into *FGFR1*^*high*^ group and *FGFR1*^*low/disomy*^ group. Then we assessed the survival outcomes of these two groups, *FGFR1*^*high*^ group did not represent a statistically significant adverse prognosis than *FGFR1*^*low/disomy*^ group, which is different from the former research [24] (Figure 3C and 3D).

The result of univariate analysis revealed that both DFS and OS were significantly associated with clinical stage (*P*<0.001), lymph node metastasis (*P*<0.001), distant metastasis (*P*<0.001), vessel involvement (*P*=0.002), and nerve involvement (DFS, *P*=0.002; OS, *P*<0.001). Upon multivariate analysis, both DFS and OS were associated with clinical stage (DFS, *P*=0.001; OS, *P*=0.003), lymph node metastasis (DFS, *P*=0.013; OS, *P*=0.009), and distant metastasis (*P*<0.001). However, no matter in univariate or multivariate analysis, *FGFR1* amplification status is not a prognostic factor concerning to DFS and OS (Supplementary Table 1).

### Survival outcomes based on clinical stage

To the stage I-II group, the estimated mean DFS time of the *FGFR1*^*high*^ group was significantly shorter compared with that of the *FGFR1*^*low/disomy*^ group (41.35±6.58 and 71.45±2.42 *P*=0.019, Figure 3E). Meanwhile, the estimated mean OS time was 47.59±5.56 and 74.10±2.27 months for patients with *FGFR1*^*high*^ and *FGFR1*^*low/disomy*^ tumors (*P*=0.034, Figure 3F). As to the stage III-IV patients, 5-year DFS and OS rate had no significant difference between *FGFR1*^*high*^ and *FGFR1*^*low/disomy*^ group. Conversely, *FGFR1*^*high*^ group showed a little better survival rate than *FGFR1*^*low/disomy*^ group (Figure 3G and 3F).

The univariate analysis revealed that patients with *FGFR1*^*high*^ had a significantly adverse DFS and OS rate (DFS, *P*=0.023; OS, *P*=0.039) than patients with *FGFR1*^*low/disomy*^ in stage I-II patients other than in stage III-IV patients. In addition, both DFS and OS were significantly associated with lymph node metastasis (*P*<0.001) and distant metastasis (*P*<0.001) in stage I-II patients and III-IV patients (Table 3 and Supplementary Table 2). However, the multivariate analysis revealed that the *FGFR1*^*high*^ is not a significant independent adverse factor for DFS and OS in stage I-II patients and III-IV patients (Table 3 and Supplementary Table 2).

**Table 3:** Association between clinicopathological characteristics and DFS/OS by COX regression model analysis in ESCC patients with stage I and II disease.

	DFS						OS					
	Univariate			Multivariate			Univariate			Multivariate		
	HR	CI (95%)	P value	HR	CI (95%)	P value	HR	CI (95%)	P value	HR	CI (95%)	P value
Gender												
Male	1			1			1			1		
Female	0.964	0.622-1.492	0.868	1.190	0.762-1.861	0.445	0.982	0.629-1.534	0.936	1.214	0.770-1.913	0.403
Age												
<60	1						1					
≥60	1.068	0.735-1.551	0.729				1.047	0.715-1.531	0.814			
Tumor site												
Upper/middle	1						1					
Low	0.920	0.631-1.340	0.663				0.930	0.632-1.369	0.712			
Smoking												
No	1						1					
Yes	0.944	0.637-1.401	0.776				1.007	0.676-1.499	0.973			
Necrosis												
No	1						1					
Yes	1.119	0.738-1.697	0.597				1.118	0.728-1.717	0.611			
Differentiation												
Well/Moderate	1			1			1			1		
Poor	1.288	0.885-1.873	0.186	1.029	0.698-1.517	0.886	1.176	0.799-1.732	0.410	0.873	0.582-1.311	0.513
Invasion depth												
I+II	1			1			1			1		
III	0.987	0.637-1.527	0.952	1.560	0.933-2.609	0.090	1.073	0.691-1.666	0.753	1.686	0.996-2.855	0.052
Lymph node metastasis												
No	1			1			1			1		
Yes	2.474	1.649-3.711	<0.001*	2.469	1.565-3.895	<0.001*	2.694	1.790-4.057	<0.001*	2.899	1.824-4.606	<0.001*
Distant metastasis												
No	1			1			1			1		
Yes	6.275	3.996-9.854	<0.001*	6.131	3.769-9.971	<0.001*	6.162	3.911-9.708	<0.001*	6.389	3.884-10.509	<0.001*
Vessel involvement												
No	1			1			1			1		
Yes	1.159	0.674-1.995	0.594	0.825	0.474-1.436	0.497	1.133	0.647-1.985	0.662	0.812	0.459-1.438	0.475
Nerve involvement												
No	1			1			1			1		
Yes	1.046	0.686-1.593	0.835	1.180	0.737-1.889	0.491	1.172	0.767-1.791	0.464	1.396	0.862-2.261	0.175
FGFR1 amplification												
Disomy/Low amplification	1			1			1			1		
High amplification	2.001	1.099-3.644	0.023*	1.646	0.900-3.008	0.106	1.933	1.035-3.610	0.039*	1.544	0.823-2.896	0.176

Invasive depth I, tumors had invaded to the mucous layer or submucosa; II, to the muscularis propria; III, to or beyond the adventitia.

CI, confidence interval; HR, hazard ratio. ^*^P<0.05 indicated that the 95% CI of HR was not including 1.

### Survival outcomes based on DFS time

As DFS time is a valuable parameter in evaluating patients’ health status after surgery and the impact of *FGFR1* amplification to DFS and OS showed a delayed trend, so we divided the whole cohort of ESCC patients into two groups according to different DFS times (24, 26, 28, 30, and 32 month respectively). After calculating these five different dividing months, we found that 30-month was the best dividing cutoff value (Supplementary Table 3). Therefore, the cohort of ESCC patients were divided into DFS<30 months group and DFS≥30 months group.

The estimated mean DFS time of the *FGFR1*^*high*^ patients was significantly shorter compared with that of the *FGFR1*^*low/disomy*^ patients in DFS≥30 months group (72.80±6.90 and 91.74±1.58 months *P*=0.009, Figure 3K). In addition, the estimated mean OS time was 74.36±6.31 and 92.59±1.49 months for patients with *FGFR1*^*high*^ and *FGFR1*^*low/disomy*^ tumors in DFS≥30 months group (*P*=0.007, Figure 3L). No matter concerning to DFS or OS, there is no difference between *FGFR1*^*high*^ and *FGFR1*^*low/disomy*^ in DFS<30 months group (Figure 3I and 3J).

The univariate analysis revealed that patients with *FGFR1*^*high*^ had a significantly adverse DFS and OS rate (DFS, *P*=0.013; OS, *P*=0.010) than patients with *FGFR1*^*low/disomy*^ in DFS≥30 months group other than in DFS<30 months group (Table 4 and Supplementary Table 4). In addition, both DFS and OS were significantly associated with lymph node metastasis (*P*<0.001), distant metastasis (*P*<0.001), and vessel involvement (*P*<0.001) in DFS≥30 months group (Table 4). However, the multivariate analysis revealed that a *FGFR1*^*high*^ is not a significant independent adverse factor for DFS and OS in DFS<30 months group and DFS≥30 months group (Table 4 and Supplementary Table 4).

**Table 4:** Association between clinicopathological characteristics and DFS/OS by COX regression model analysis in ESCC patients with DFS≥30 months.

	DFS									OS								
	Univariate					Multivariate				Univariate					Multivariate			
	HR		CI (95%)	P value		HR		CI (95%)	P value	HR	CI (95%)			P value	HR	CI (95%)		P value	
Gender																			
Male	1					1				1					1				
Female	1.352		0.682-2.683	0.388		1.664		0.818-3.384	0.160	1.308	0.641-2.668			0.461	1.626	0.776-3.404		0.198	
Age																			
<60	1									1									
≥60	0.965		0.530-1.758	0.908						0.891	0.483-1.645			0.713					
Tumor site																			
Upper/middle	1									1									
Low	1.072		0.589-1.953	0.820						1.114	0.603-2.058			0.731					
Smoking																			
No	1									1									
Yes	0.974		0.520-1.823	0.934						1.153	0.615-2.159			0.657					
Necrosis																			
No	1									1									
Yes	1.261		0.657-2.423	0.486						1.434	0.742-2.772			0.284					
Differentiation																			
Well/Moderate	1					1				1					1				
Poor	1.701		0.935-3.093	0.082		1.014		0.521-1.972	0.968	1.857	1.006-3.427			0.048*	1.021	0.514-2.028		0.953	
Clinical stage																			
I+II	1					1				1					1				
III	2.563		1.390-4.726	0.003*		0.428		0.142-1.292	0.132	2.739	1.471-5.100			0.001*	0.529	0.178-1.574		0.253	
Invasion depth																			
I+II	1					1				1					1				
III+IV	1.031		0.545-1.953	0.925		1.148		0.471-2.802	0.761	0.928	0.480-1.792			0.823	0.814	0.320-2.071		0.665	
Lymph node metastasis																			
No	1					1				1					1				
Yes	3.109		1.702-5.678	<0.001*		3.304		1.293-8.441	0.013*	3.400	1.826-6.331			<0.001*	3.053	1.193-7.815		0.020*	
Distant metastasis																			
No	1					1				1					1				
Yes	12.682		6.145-26.174	<0.001*		11.347		4.390-29.329	<0.001*	10.692	5.166-22.127			<0.001*	7.802	3.077-19.781		<0.001*	
Vessel involvement																			
No	1					1				1					1				
Yes	3.088		1.675-5.693	<0.001*		1.999		0.973-4.107	0.059	3.559	1.919-6.599			<0.001*	2.389	1.157-4.934		0.019*	
Nerve involvement																			
No	1					1				1					1				
Yes	1.221		0.645-2.312	0.540		1.393		0.638-3.044	0.405	1.306	0.685-2.490			0.418	1.627	0.721-3.670		0.241	
FGFR1 amplification																			
Disomy/Low amplification	1					1				1					1				
High amplification	2.789	1.239-6.276		0.013*		1.490		0.614-3.620		0.378	2.896		1.283-6.536		0.010*	1.515	0.620-3.705	0.362		

Invasive depth I, tumors had invaded to the mucous layer or submucosa; II, to the muscularis propria; III, to or beyond the adventitia.

CI, confidence interval; HR, hazard ratio. ^*^P<0.05 indicated that the 95% CI of HR was not including 1.

### Survival outcomes based on clinical stage and DFS time

**Figure 4:**
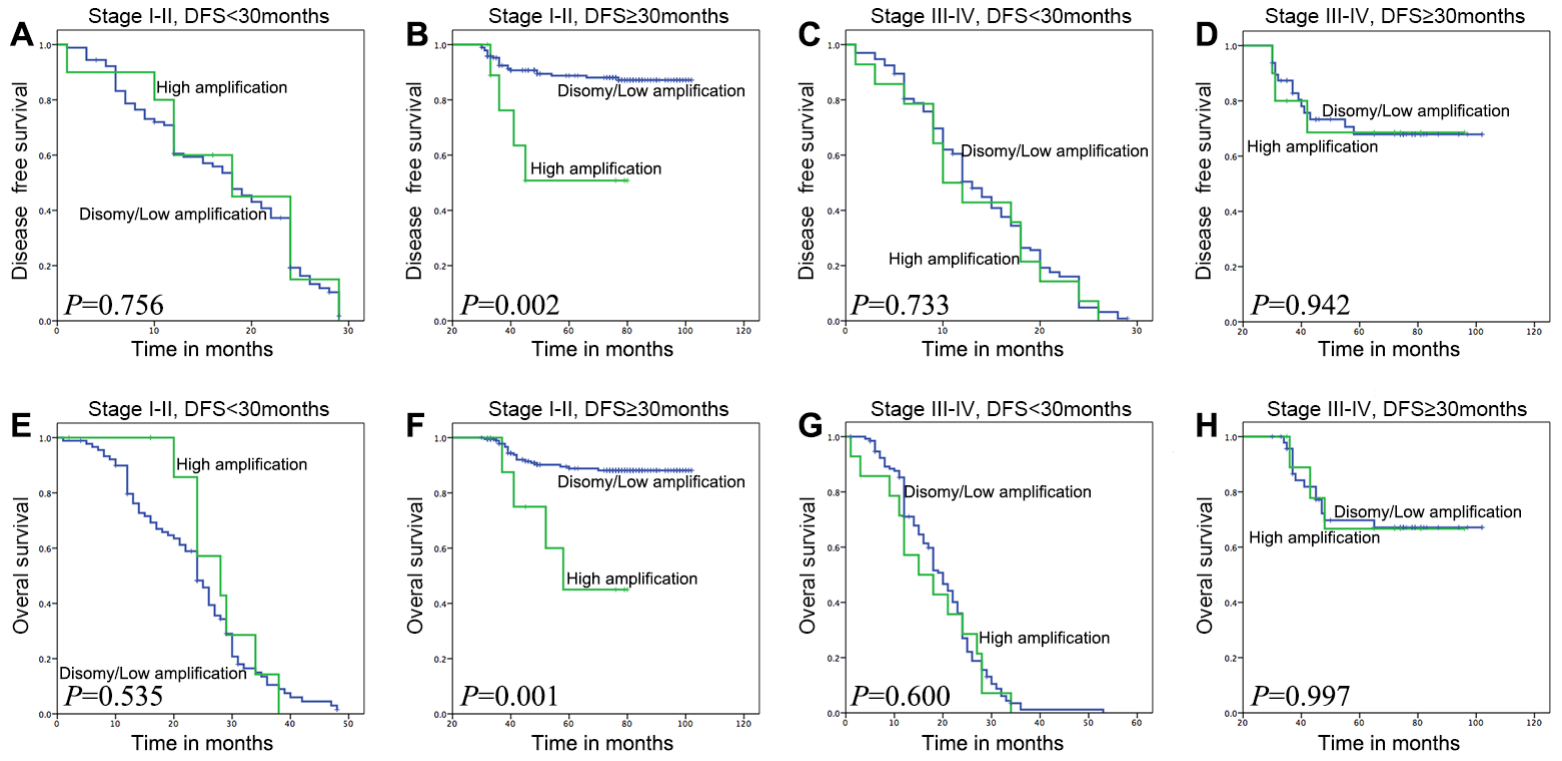
Survival analyses based on clinical stage and DFS time 30 months of ESCC patients In stage I-II patients with DFS time≥30 months, high *FGFR1* amplification was associated with adverse DFS and OS (**B and F**; *P*=0.002 or 0.001), but not in other three groups which are stage I-II with DFS<30 months, stage III-IV with DFS<30 months, and stage III-IV with DFS≥30 months (DFS: **A, C and D**; *P*=0.756, 0.733 or 0.942; OS: **E, G and H**; *P*=0.535, 0.600 or 0.997).

We combined the clinical stage and DFS 30-month to divide the whole cohort of ESCC patients into 4 groups. To the subset of patients whose DFS≥30 months in stage I-II, *FGFR1*^*high*^ showed a significant adverse influence to both DFS and OS (*P*=0.002 and *P*=0.003, Figure 4B and 4F). And there was no difference between *FGFR1*^*high*^ and *FGFR1*^*low/disomy*^ groups in other three subsets (Figure 4A, 4C-4E, 4G-4H).

To the subset of patients whose DFS time≥30 months in stage I-II, the results of the univariate analysis revealed that *FGFR1*^*high*^ group had a significantly shorter DFS and OS time (DFS, *P*=0.006; OS, *P*=0.003) than patients with *FGFR1*^*low/disomy*^ (Table 5). In addition, both DFS and OS were significantly associated with lymph node metastasis (DFS, *P*=0.002; OS, *P*=0.001) and distant metastasis (*P*<0.001, Table 5). The multivariate analysis showed that *FGFR1*^*high*^ was significantly associated with a shorter OS (*P*=0.037, Table 5). However, there was no trend toward worse DFS for *FGFR1*^*high*^ comparing to *FGFR1*^*low/disomy*^ in multivariate analysis (*P*=0.123, Table 5)

**Table 5:** Association between clinicopathological characteristics and DFS/OS by COX regression model analysis in ESCC patients with DFS≥30 months in stage I and II.

	DFS						OS					
	Univariate			Multivariate			Univariate			Multivariate		
	HR	CI (95%)	P value	HR	CI (95%)	P value	HR	CI (95%)	P value	HR	CI (95%)	P value
Gender												
Male	1			1			1			1		
Female	1.536	0.668-3.533	0.313	2.498	1.017-6.136	0.046*	1.795	0.768-4.194	0.177	2.859	1.131-7.227	0.026*
Age												
<60	1						1					
≥60	0.663	0.307-1.434	0.297				0.559	0.248-1.259	0.161			
Tumor site												
Upper/middle	1						1					
Low	0.830	0.370-1.864	0.653				0.833	0.356-1.947	0.673			
Smoking												
No	1						1					
Yes	0.637	0.256-1.587	0.333				0.688	0.273-1.732	0.427			
Necrosis												
No	1						1					
Yes	0.840	0.316-2.231	0.726				0.989	0.369-2.653	0.983			
Differentiation												
Well/Moderate	1			1			1			1		
Poor	1.345	0.610-2.965	0.462	1.032	0.442-2.409	0.942	1.273	0.557-2.909	0.567	0.785	0.304-1.888	0.552
Invasion depth												
I+II	1			1			1			1		
III	0.784	0.295-2.081	0.625	1.080	0.313-3.728	0.904	0.819	0.306-2.194	0.691	0.885	0.242-3.234	0.854
Lymph node metastasis												
No	1			1			1			1		
Yes	3.635	1.580-8.366	0.002*	5.211	1.896-14.317	0.001*	4.021	1.720-9.401	0.001*	5.164	1.878-14.195	0.001*
Distant metastasis												
No	1			1			1			1		
Yes	19.124	7.023-52.075	<0.001*	30.942	9.189-104.191	<0.001*	14.484	5.255-39.919	<0.001*	20.623	6.180-68.817	<0.001*
Vessel involvement												
No	1			1			1			1		
Yes	2.432	0.976-6.059	0.056	2.154	0.777-5.973	0.140	2.608	1.035-6.547	0.042*	2.343	0.863-6.363	0.095
Nerve involvement												
No	1			1			1			1		
Yes	1.234	0.518-2.938	0.635	1.885	0.629-5.646	0.258	1.424	0.590-3.435	0.431	2.653	0.823-8.557	0.102
FGFR1 amplification												
Disomy/Low amplification	1			1			1			1		
High amplification	4.521	1.549-13.195	0.006*	2.430	0.786-7.514	0.123	5.180	1.766-15.194	0.003*	3.239	1.076-9.746	0.037*

Invasive depth I, tumors had invaded to the mucous layer or submucosa; II, to the muscularis propria; III, to or beyond the adventitia.

CI, confidence interval; HR, hazard ratio. ^*^P<0.05 indicated that the 95% CI of HR was not including 1.

## DISCUSSION

The poor prognosis of ESCC highlights the need for new prognostic markers in this disease and an improved understanding of the key genetic and progression are critical for the development of effective therapeutics.

In the present study, we investigated whether high *FGFR1* amplification was associated with the clinicopathological parameters and its impact on survival in patients with operable ESCC. Our study demonstrated that high *FGFR1* amplification is not a common genetic alteration (8.7%) but presented as a delayed adverse prognostic factor in resected stage I-II ESCC patients. To the best of our knowledge, this is the first report analyzing the prognosis impact of *FGFR1* amplification by linking it to clinical stage and DFS time. This information could have clinical value in identifying ESCC patients who are at high risk of progression and may benefit the most from FGFR-inhibiting drugs.

The prevalence of *FGFR1* amplification is the firstly determining factor in judging whether FGFR inhibitors could be used in ESCC patients. Different from a singular criterion only using *FGFR1* copy numbers, we chose a more sophisticated *FGFR1* FISH criterion considering gene copy number per nucleus, *FGFR1*/CEN8 ratio, and percentage of gene clusters at the same time [30]. Among those patients, 8.7% were high *FGFR1* amplification, 3.6% were low *FGFR1* amplification, and 87.7% were disomy. The *FGFR1* amplification rate (12.3%) of our study, as determined by FISH analysis, was comparable to previous reports, ranging from 6% to 9.7%[27-29]. Given the relatively specific *FGFR1* amplification, it may represent a potential therapeutic target for ESCC.

Recently, genome-wide comprehensive analysis found considerable overlap between the genes that are frequently mutated in ESCC and other types of squamous cell carcinoma [6, 7]. *FGFR1* amplification has been reported in numerous kinds of tumors, especially in squamous cell lung cancer [13, 20] and head and neck squamous cell carcinoma [22]. However, when considering the prognostic value of *FGFR1* amplification, the results are controversial. In resected squamous cell lung cancer, one study found that no significant difference in OS by *FGFR1* amplification status no matter in the whole research group or in advanced stage subset [31]. In another study, *FGFR1* amplification is an independent negative prognostic factor in surgically resected squamous cell lung cancer [19]. And the prognostic value is not consistent in ESCC between different groups as whether high *FGFR1* amplification is an independent poor prognostic factor or no association between *FGFR1* amplification status and clinical outcome [27, 28]. This is may be due to different races and different criteria.

In our study, we evaluate the estimated mean DFS times and OS times in three *FGFR1* groups (high amplification, low *FGFR1* amplification and disomy), and the DFS and OS time has no significant difference which is consistent with Kim’s report [27]. Due to the similar survival outcome between low and no *FGFR1* amplification group, we categorized total patients into *FGFR1*^*high*^ group and *FGFR1*^*low/disomy*^ group and assessed the survival outcomes of these two groups. *FGFR1*^*high*^ did not represent a statistically significant adverse prognosis, which is different from the previous report [27], and this is may be owing to the tumor heterogeneity of different research groups from different countries.

As clinical stage is an important clinicopathological feature, the prognosis usually vary in patients with different stages. To squamous cell lung cancer, the *FGFR1* amplification is an independent adverse prognostic marker in early stage patients [32], and another study found that no significant difference in OS by *FGFR1* amplification status in advanced stage subset [31]. We then categorized the patients into stage I-II group and stage III-IV group. To the stage I-II group, the estimated mean DFS and OS time of the *FGFR1*^*high*^ group was significantly shorter compared with that of the *FGFR1*^*low/disomy*^ group. Interesting, the trend is only can be detected in stage I-II group other than stage III-IV group, implying that high *FGFR1* amplification prognostic value is relying on clinical stage. The result is confirmed by univariate COX regression analysis.

DFS time is measured from the time of surgery to initial tumor relapse (local recurrence or distant) or death as a result of any cause and is an important index for surgeon to follow up. Moreover, DFS is also served as the endpoint of the phase III clinical trials of antitumor drugs. When we divided the whole group into two subsets according to the DFS time (30-month), the estimated mean DFS and OS time of the *FGFR1*^*high*^ patients was significantly shorter compared with that of the *FGFR1*^*low/disomy*^ patients in DFS≥30 months group. Clearly, after the patient finished radical operation, if the patient can live without recurrence or distant metastasis for more than 30 months, the *FGFR1*^*high*^ status is important to the prognosis of the patient, indicating that high *FGFR1* amplification is a delayed prognostic factor which is just as same as the prognostic value of EGFR amplification in ESCC [33].

Aimed to find the most influenced subgroup patients by *FGFR1*^*high*^ status, we firstly combined the clinical stage and DFS 30-month to divide the whole cohort of ESCC patients. To the subset of patients whose DFS≥30 months in stage I-II, *FGFR1*^*high*^ showed a significant adverse influence to both DFS and OS. And there is no difference between *FGFR1*^*high*^ and *FGFR1*^*low/disomy*^ groups in other three subsets. By this way, we precisely selected the most influenced patients by *FGFR1*^*high*^ status.

*FGFR1* amplification is one of the most promising findings in ESCC due to the availability of FGFR inhibitors’ application. Several small molecules, such AZD4547 and PD173073, targeting the *FGFR1* tyrosine kinase are now in clinical trials for the treatment of patients with squamous cell lung cancer and other solid malignant tumors [13, 34]. And *FGFR1* amplification is the most important inclusion criterion. Our study precisely stratified the patients according to clinical stage and DFS time, finding out the subgroup patients whose prognosis were most decreased by high *FGFR1* amplification and this may be important in the future for selecting patients enrolled in clinical trials, providing a theoretical basis in patient selection.

In conclusion, we demonstrated that *FGFR1* amplification was an infrequent genetic alteration, and for firstly, we found high *FGFR1* amplification was an independent delayed adverse prognostic factor only in stage I-II ESCC patients, suggesting that FGFR amplification may be a viable prognostic factor in these patients.

## MATERIALS AND METHODS

### Patients and tissue samples

The study was conducted in a cohort of patients with ESCC who underwent surgical resection in our institution from 2007 to 2010. Two pathologists confirmed the diagnosis of ESCC by hematoxylin-eosin (HE) staining. A predesigned data collection format was used to review the patients’ medical records for evaluation of clinicopathological characteristics and survival outcomes. We identified 592 patients. Of those we excluded patients with very limited tumor tissue, patients with neoadjuvant therapy, and patients with incomplete clinicopathological information or survival information (n=49). Paraffin-embedded (FFPE) tumor specimens were used to construct a tissue microarray (TMA) [35] and then performed HE staining to estimate the tumor ratio of each core. A total of 37 patients were excluded because of limited tumor tissue (less than 100 tumor nuclei) in TMA (Figure 1, Table 1). Eventually, the tumor samples of 506 patients were available for examination of *FGFR1* amplification. Each patient provided informed consent for the use of their tissue samples and the study was approved by the institutional review board of Zhongshan Hospital.

### *FGFR1* fluorescence in situ hybridization

Fluorescent in situ hybridization (FISH) assay was performed on the tissue microarrays by using *FGFR1* probe that hybridizes to the band 8p12 with Spectrum Red and CEP 8 with SpectrumAqua Probe RUO (green) (Abbott Molecular, Abbott Park, IL) following routine methods. Two experienced evaluators blinded to the clinical data interpreted FISH analyses. At least 100 nuclei per patient were evaluated. The prespecified threshold for assigning a sample to the *FGFR1*^*high*^ group was an *FGFR1*/CEN8 ratio is ≥ 2.0, or average number of *FGFR1* signals/ tumor cell nucleus ≥ 6.0, or percentage of tumor cells containing ≥ 15 *FGFR1* signals or large cluster in ≥ 10%. Percentage of tumor cells containing ≥ 5 *FGFR1* signals in ≥ 50% was defined as low amplification, and a copy number of two was considered disomy [30].

### Statistical analysis

The primary end point was to assess whether *FGFR1* amplification affected survival in terms of DFS and OS in patients with resected ESCC. DFS was measured from the time of surgery to initial tumor relapse (local recurrence or distant) or death as a result of any cause. OS, calculated from the time of surgery to death or last follow-up date. Associations with clinical characteristics were evaluated by using Fisher’s exact test or the χ^2^ test. Kaplan-Meier curves with log-rank tests were used to calculate the cumulative survival proportion for OS and DFS by *FGFR1* amplification level. A Cox proportional hazards model was applied to investigate the univariate and multivariate hazard ratios for the study variables. Multivariate analysis was performed for all the significant variables in the univariate analysis. Statistical significance was set at *P*<0.05 for all analyses. All statistical analyses were performed by using SPSS version 22.0 (SPSS, Chicago, IL).

## SUPPLEMENTARY MATERIALS TABLES










