# Supplementary material for: High amplification of FGFR1 gene is a delayed poor prognostic factor in early stage ESCC patients
Source: Oncotarget. 2017 Aug 12;8(43):74539–53. doi: 10.18632/oncotarget.20215 (PMC5650361; doi:10.18632/oncotarget.20215)
Supplement: Supplementary file 1 [file oncotarget-08-74539-s001.pdf]

High amplification of FGFR1 gene is a delayed poor prognostic factor in early stage ESCC patients

SUPPLEMENTARY MATERIALS

Supplementary Table 1: Association between clinicopathological characteristics and DFS/OS by COX regression model analysis in this cohort of 506 ESCC patients.

See Supplementary File 1

Supplementary Table 2: Association between clinicopathological characteristics and DFS/OS by COX regression model analysis in ESCC patients with stage III and IV disease.

See Supplementary File 2

Supplementary Table 3: Kaplan–Meier analysis for DFS and OS (divided patients by a series of DFS times)

See Supplementary File 3

Supplementary Table 4: Association between clinicopathological characteristics and DFS/OS by COX regression model analysis in ESCC patients with DFS time less than 30 months.

See Supplementary File 4
