# Supplementary material for: High amplification of FGFR1 gene is a delayed poor prognostic factor in early stage ESCC patients
Source: Oncotarget. 2017 Aug 12;8(43):74539–53. doi: 10.18632/oncotarget.20215 (PMC5650361; doi:10.18632/oncotarget.20215)
Supplement: Supplementary file 2 [file oncotarget-08-74539-s002.docx]

| Supplementary Table 1: Association between clinicopathological characteristics and DFS/OS by COX regression model analysis in this cohort of 506 ESCC patients. | | | | | | | | | | | | | | | |
| --- | --- | --- | --- | --- | --- | --- | --- | --- | --- | --- | --- | --- | --- | --- | --- |
|  | DFS | | | | | | |  | OS | | | | | | |
|  | Univariate | | |  | Multivariate | | |  | Univariate | | |  | Multivariate | | |
|  | HR | CI (95%) | P value |  | HR | CI (95%) | P value |  | HR | CI (95%) | P value |  | HR | CI (95%) | P value |
| Gender |  |  |  |  |  |  |  |  |  |  |  |  |  |  |  |
| Male | 1 |  |  |  | 1 |  |  |  | 1 |  |  |  | 1 |  |  |
| Female | 0.810 | 0.591-1.109 | 0.188 |  | 1.042 | 0.755-1.439 | 0.802 |  | 0.754 | 0.542-1.049 | 0.094 |  | 0.985 | 0.702-1.381 | 0.929 |
| Age |  |  |  |  |  |  |  |  |  |  |  |  |  |  |  |
| <60 | 1 |  |  |  |  |  |  |  | 1 |  |  |  |  |  |  |
| ≥60 | 1.010 | 0.795-1.284 | 0.933 |  |  |  |  |  | 1.001 | 0.785-1.277 | 0.993 |  |  |  |  |
| Tumor site |  |  |  |  |  |  |  |  |  |  |  |  |  |  |  |
| Upper/middle | 1 |  |  |  |  |  |  |  | 1 |  |  |  |  |  |  |
| Low | 1.046 | 0.825-1.327 | 0.710 |  |  |  |  |  | 1.082 | 0.849-1.378 | 0.524 |  |  |  |  |
| Nerve involvement |  |  |  |  |  |  |  |  |  |  |  |  |  |  |  |
| No | 1 |  |  |  | 1 |  |  |  | 1 |  |  |  | 1 |  |  |
| Yes | 1.457 | 1.145-1.853 | 0.002* |  | 1.109 | 0.847-1.451 | 0.452 |  | 1.546 | 1.210-1.975 | <0.001* |  | 1.204 | 0.915-1.584 | 0.185 |
| Smoking |  |  |  |  |  |  |  |  |  |  |  |  |  |  |  |
| No | 1 |  |  |  |  |  |  |  | 1 |  |  |  |  |  |  |
| Yes | 1.121 | 0.880-1.428 | 0.353 |  |  |  |  |  | 1.194 | 0.934-1.525 | 0.157 |  |  |  |  |
| Differentiation |  |  |  |  |  |  |  |  |  |  |  |  |  |  |  |
| Well/Moderate | 1 |  |  |  | 1 |  |  |  | 1 |  |  |  | 1 |  |  |
| Poor | 1.279 | 1.007-1.624 | 0.043* |  | 0.928 | 0.721-1.195 | 0.561 |  | 1.221 | 0.957-1.558 | 0.109 |  | 0.874 | 0.676-1.130 | 0.304 |
| Clinical stage |  |  |  |  |  |  |  |  |  |  |  |  |  |  |  |
| I+II | 1 |  |  |  | 1 |  |  |  | 1 |  |  |  | 1 |  |  |
| III+IV | 3.235 | 2.536-4.127 | <0.001* |  | 2.001 | 1.319-3.036 | 0.001* |  | 3.356 | 2.618-4.301 | <0.001* |  | 1.943 | 1.261-2.992 | 0.003* |
| Invasion depth |  |  |  |  |  |  |  |  |  |  |  |  |  |  |  |
| I+II | 1 |  |  |  | 1 |  |  |  | 1 |  |  |  | 1 |  |  |
| III | 1.180 | 0.921-1.511 | 0.190 |  | 1.063 | 0.789-1.432 | 0.687 |  | 1.198 | 0.931-1.542 | 0.161 |  | 1.039 | 0.765-1.413 | 0.805 |
| Lymph node metastasis |  |  |  |  |  |  |  |  |  |  |  |  |  |  |  |
| No | 1 |  |  |  | 1 |  |  |  | 1 |  |  |  | 1 |  |  |
| Yes | 3.059 | 2.376-3.937 | <0.001* |  | 1.683 | 1.116-2.539 | 0.013* |  | 3.197 | 2.470-4.139 | <0.001* |  | 1.758 | 1.148-2.692 | 0.009* |
| Distant metastasis |  |  |  |  |  |  |  |  |  |  |  |  |  |  |  |
| No | 1 |  |  |  | 1 |  |  |  | 1 |  |  |  | 1 |  |  |
| Yes | 3.849 | 2.868-5.165 | <0.001* |  | 3.363 | 2.417-4.679 | <0.001* |  | 3.706 | 2.758-4.980 | <0.001* |  | 3.205 | 2.302-4.464 | <0.001* |
| Vessel involvement |  |  |  |  |  |  |  |  |  |  |  |  |  |  |  |
| No | 1 |  |  |  | 1 |  |  |  | 1 |  |  |  | 1 |  |  |
| Yes | 1.517 | 1.166-1.976 | 0.002* |  | 0.847 | 0.635-1.129 | 0.257 |  | 1.539 | 1.176-2.014 | 0.002* |  | 0.870 | 0.650-1.165 | 0.351 |
| Necrosis |  |  |  |  |  |  |  |  |  |  |  |  |  |  |  |
| No | 1 |  |  |  |  |  |  |  | 1 |  |  |  |  |  |  |
| Yes | 1.234 | 0.959-1.589 | 0.102 |  |  |  |  |  | 1.211 | 0.934-1.570 | 0.148 |  |  |  |  |
| *FGFR1* amplification |  |  |  |  |  |  |  |  |  |  |  |  |  |  |  |
| Disomy/Low amplification | 1 |  |  |  | 1 |  |  |  | 1 |  |  |  | 1 |  |  |
| High amplification | 1.362 | 0.927-2.003 | 0.116 |  | 0.947 | 0.638-1.406 | 0.786 |  | 1.367 | 0.924-2.023 | 0.118 |  | 0.968 | 0.646-1.449 | 0.873 |
| Invasive depth I, tumors had invaded to the mucous layer or submucosa; II, to the muscularis propria; III, to or beyond the adventitia.  CI, confidence interval; HR, hazard ratio. ^*^ P<0.05 indicated that the 95% CI of HR was not including 1 | | | | | | | | | | | | | | | |
