# Supplementary material for: High amplification of FGFR1 gene is a delayed poor prognostic factor in early stage ESCC patients
Source: Oncotarget. 2017 Aug 12;8(43):74539–53. doi: 10.18632/oncotarget.20215 (PMC5650361; doi:10.18632/oncotarget.20215)
Supplement: Supplementary file 4 [file oncotarget-08-74539-s004.docx]

| Supplementary Table 3: Kaplan–Meier analysis for DFS and OS (divided patients by a series of DFS times) | | | | | | | | | | |
| --- | --- | --- | --- | --- | --- | --- | --- | --- | --- | --- |
|  | 24-month | | 26-month | | 28-month | | 30-month | | 32-month | |
|  | < | ≥ | < | ≥ | < | ≥ | < | ≥ | < | ≥ |
| DFS | 0.938 | 0.032 | 0.825 | 0.011 | 0.904 | 0.015 | 0.964 | 0.009 | 0.726 | 0.025 |
| OS | 0.258 | 0.014 | 0.767 | 0.006 | 0.839 | 0.011 | 0.912 | 0.007 | 0.625 | 0.019 |
