# Supplementary material for: High amplification of FGFR1 gene is a delayed poor prognostic factor in early stage ESCC patients
Source: Oncotarget. 2017 Aug 12;8(43):74539–53. doi: 10.18632/oncotarget.20215 (PMC5650361; doi:10.18632/oncotarget.20215)
Supplement: Supplementary file 5 [file oncotarget-08-74539-s005.docx]

| Supplementary table 4：Association between clinicopathological characteristics and DFS/OS by COX regression model analysis in ESCC patients with DFS time less than 30 months. | | | | | | | | | | | | | | | |
| --- | --- | --- | --- | --- | --- | --- | --- | --- | --- | --- | --- | --- | --- | --- | --- |
|  | DFS | | | | | | |  | OS | | | | | | |
|  | Univariate | | |  | Multivariate | | |  | Univariate | | |  | Multivariate | | |
|  | HR | CI (95%) | P value |  | HR | CI (95%) | P value |  | HR | CI (95%) | P value |  | HR | CI (95%) | P value |
| Gender |  |  |  |  |  |  |  |  |  |  |  |  |  |  |  |
| Male | 1 |  |  |  | 1 |  |  |  | 1 |  |  |  | 1 |  |  |
| Female | 0.638 | 0.445-0.914 | 0.014* |  | 0.664 | 0.456-0.968 | 0.033* |  | 0.646 | 0.443-0.942 | 0.023* |  | 0.663 | 0.448-0.981 | 0.040* |
| Age |  |  |  |  |  |  |  |  |  |  |  |  |  |  |  |
| <60 | 1 |  |  |  |  |  |  |  | 1 |  |  |  |  |  |  |
| ≥60 | 0.795 | 0.611-1.033 | 0.086 |  |  |  |  |  | 0.955 | 0.731-1.248 | 0.735 |  |  |  |  |
| Tumor site |  |  |  |  |  |  |  |  |  |  |  |  |  |  |  |
| Upper/middle | 1 |  |  |  |  |  |  |  | 1 |  |  |  |  |  |  |
| Low | 1.017 | 0.784-1.318 | 0.900 |  |  |  |  |  | 1.099 | 0.843-1.433 | 0.486 |  |  |  |  |
| Nerve involvement |  |  |  |  |  |  |  |  |  |  |  |  |  |  |  |
| No | 1 |  |  |  | 1 |  |  |  | 1 |  |  |  | 1 |  |  |
| Yes | 0.982 | 0.757-1.275 | 0.893 |  | 0.789 | 0.591-1.053 | 0.108 |  | 1.194 | 0.912-1.564 | 0.198 |  | 0.966 | 0.715-1.306 | 0.824 |
| Smoking |  |  |  |  |  |  |  |  |  |  |  |  |  |  |  |
| No | 1 |  |  |  |  |  |  |  | 1 |  |  |  |  |  |  |
| Yes | 1.008 | 0.775-1.311 | 0.952 |  |  |  |  |  | 1.030 | 0.788-1.346 | 0.829 |  |  |  |  |
| Differentiation |  |  |  |  |  |  |  |  |  |  |  |  |  |  |  |
| Well/Moderate | 1 |  |  |  | 1 |  |  |  | 1 |  |  |  | 1 |  |  |
| Poor | 1.055 | 0.813-1.371 | 0.686 |  | 0.952 | 0.725-1.250 | 0.723 |  | 0.942 | 0.720-1.232 | 0.661 |  | 0.843 | 0.638-1.114 | 0.230 |
| Clinical stage |  |  |  |  |  |  |  |  |  |  |  |  |  |  |  |
| I+II | 1 |  |  |  | 1 |  |  |  | 1 |  |  |  | 1 |  |  |
| III+IV | 1.625 | 1.238-2.133 | <0.001* |  | 1.843 | 1.225-2.772 | 0.003* |  | 1.652 | 1.253-2.178 | <0.001* |  | 1.500 | 0.966-2.329 | 0.071 |
| Invasion depth |  |  |  |  |  |  |  |  |  |  |  |  |  |  |  |
| I+II | 1 |  |  |  | 1 |  |  |  | 1 |  |  |  | 1 |  |  |
| III | 0.991 | 0.757-1.297 | 0.948 |  | 1.150 | 0.841-1.573 | 0.382 |  | 1.270 | 0.962-1.675 | 0.091 |  | 1.447 | 1.044-2.007 | 0.027* |
| Lymph node metastasis |  |  |  |  |  |  |  |  |  |  |  |  |  |  |  |
| No | 1 |  |  |  | 1 |  |  |  | 1 |  |  |  | 1 |  |  |
| Yes | 1.424 | 1.076-1.885 | 0.013* |  | 0.982 | 0.657-1.467 | 0.930 |  | 1.462 | 1.098-1.946 | 0.009* |  | 1.066 | 0.690-1.646 | 0.773 |
| Distant metastasis |  |  |  |  |  |  |  |  |  |  |  |  |  |  |  |
| No | 1 |  |  |  | 1 |  |  |  | 1 |  |  |  | 1 |  |  |
| Yes | 1.628 | 1.183-2.243 | 0.003* |  | 1.822 | 1.275-2.603 | 0.001* |  | 1.432 | 1.039-1.973 | 0.028* |  | 1.764 | 1.231-2.528 | 0.002* |
| Vessel involvement |  |  |  |  |  |  |  |  |  |  |  |  |  |  |  |
| No | 1 |  |  |  | 1 |  |  |  | 1 |  |  |  | 1 |  |  |
| Yes | 1.080 | 0.804-1.452 | 0.609 |  | 0.894 | 0.647-1.233 | 0.494 |  | 1.139 | 0.839-1.547 | 0.403 |  | 0.950 | 0.685-1.317 | 0.758 |
| Necrosis |  |  |  |  |  |  |  |  |  |  |  |  |  |  |  |
| No | 1 |  |  |  |  |  |  |  | 1 |  |  |  |  |  |  |
| Yes | 1.254 | 0.951-1.653 | 0.108 |  |  |  |  |  | 1.150 | 0.866-1.526 | 0.334 |  |  |  |  |
| *FGFR1* amplification |  |  |  |  |  |  |  |  |  |  |  |  |  |  |  |
| Disomy/Low amplification | 1 |  |  |  | 1 |  |  |  | 1 |  |  |  | 1 |  |  |
| High amplification | 1.009 | 0.650-1.567 | 0.967 |  | 1.085 | 0.689-1.710 | 0.725 |  | 0.976 | 0.622-1.532 | 0.916 |  | 1.005 | 0.631-1.601 | 0.982 |
| Invasive depth I, tumors had invaded to the mucous layer or submucosa; II, to the muscularis propria; III, to or beyond the adventitia.  CI, confidence interval; HR, hazard ratio. ^*^ P<0.05 indicated that the 95% CI of HR was not including 1 | | | | | | | | | | | | | | | |
